# Ultrathin two-dimensional hybrid perovskites toward flexible electronics and optoelectronics

**DOI:** 10.1093/nsr/nwab129

**Published:** 2021-07-19

**Authors:** Junran Zhang, Xuefen Song, Lin Wang, Wei Huang

**Affiliations:** Key Laboratory of Flexible Electronics and Institute of Advanced Materials, School of Physical and Mathematical Sciences, Nanjing Tech University, China; Key Laboratory of Flexible Electronics and Institute of Advanced Materials, School of Physical and Mathematical Sciences, Nanjing Tech University, China; Key Laboratory of Flexible Electronics and Institute of Advanced Materials, School of Physical and Mathematical Sciences, Nanjing Tech University, China; Key Laboratory of Flexible Electronics and Institute of Advanced Materials, School of Physical and Mathematical Sciences, Nanjing Tech University, China; Frontiers Science Center for Flexible Electronics, Key Laboratory of Flexible Electronics, Shaanxi Institute of Flexible Electronics, Institute of Flexible Electronics, Northwestern Polytechnical University, China

## Abstract

Ultrathin hybrid perovskites combine the advantages of 2D morphology and organic-inorganic components. This perspective article provides an updated summary and new insights for their development in flexible electronics and optoelectronics.

The past decade has witnessed an explosion of research into organic-inorganic hybrid perovskites (OIHPs) due to their outstanding properties, including high flexibility, high absorption/emission efficiency, large defect tolerance and long-distance carrier diffusion [1]. Recently, the ultrathin two-dimensional (2D) OIHP (Fig. 1), combining the attractive features of hybrid components and 2D morphology, can be considered a rising star among novel flexible materials. Accordingly, it has been developed by a variety of synthesis approaches, such as solution processed growth [2], mechanical exfoliation [3] and chemical vapor deposition [4]. Compared with bulky counterparts, ultrathin OIHPs possess specific advantages due to their strong quantum confinement effect, such as enhanced photoluminescence quantum yield, lower thresholds for amplified spontaneous emission, easily tunable bandgaps, high exciton binding energy, strong thickness dependence and high integration and flexibility for devices [5]. Ultrathin OIHPs also provide an excellent platform for defect regulation and surface functionalization, as most atoms are exposed to the external environment [2,5]. In addition, thanks to their ultra-smooth surface and strong interface coupling, 2D heterostructures of ultrathin OIHPs can be flexibly designed without considering the lattice mismatch or growth feasibility, thus revealing new physics and functionalities [4,6].

**Figure 1.**
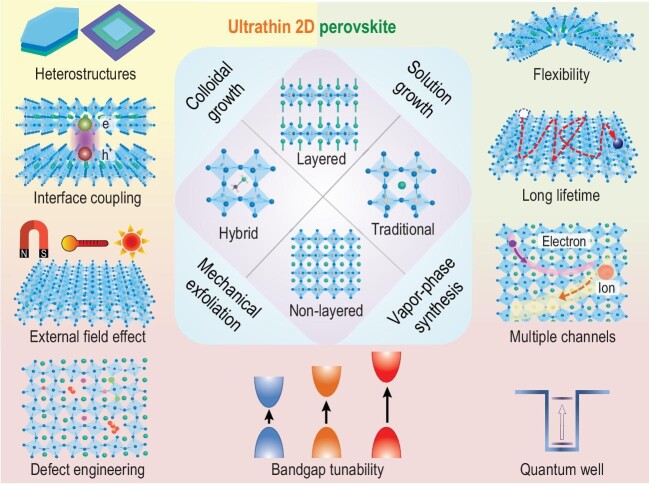
Unique features of ultrathin OIHP materials, combining the advantages of 2D morphology (e.g. heterostructures, interface coupling and external field effect) and hybrid perovskite components (e.g. flexibility, long lifetime and multiple channels) with good potential in defect engineering, bandgap tunability, quantum-well like devices etc.

Ultrathin OIHPs are unique mainly due to the coexistence of organic and inorganic atoms. They possess excellent optoelectronic properties, high sensitivity and easy processing in contrast with 2D inorganic materials, such as transition metal dichalcogenides [5]. Compared to 2D organic films, ultrathin OIHPs with rich variety, tunable structures and heavy atoms, show many interesting physical properties, for instance, in the optoelectronic, spintronic and ferroelectric fields [1,5]. It is worth noting that the electrical properties of ultrathin OIHPs are particular, and are simultaneously affected by ion migration and electron transport in 2D channels [5,7]. In short, ultrathin OIHPs containing an organic component in the molecularly thin 2D scale make themselves more promising in flexible electronics and optoelectronics [5].

To date, tremendous progress in flexible devices based on OIHP films has been achieved, such as light-emitting diodes (LEDs), photodetectors, solar cells and lasers (Fig. 2a), however, which is not the case for ultrathin OIHPs. As we know, OIHPs are promising light-emitting materials for LED applications because of their ultrahigh photoluminescence quantum efficiency [1]. Utilizing transparent and flexible electrodes/substrates, flexible LEDs based on OIHP thin films are achieved [8]. In addition, most LEDs based on OIHPs operate in infrared or long visible wavelength ranges [1]. In future investigations, it may be possible to realize mid-and-far infrared LEDs by using ultrathin OIHPs according to their excellent bandgap tunability via thickness and doping engineering [5]. Moreover, using single-crystalline film as the active layer, a flexible photodetector achieves record-high photoelectric responsivity and broadband photodetection [9]. Flexible perovskite solar cells that are promising for wearable and portable electronics, with high power conversion efficiency and open voltage and small efficiency degradation, are suitable for mass production by roll-to-roll fabrication techniques [8]. However, it is the poor charge transport occurring in the interfaces of hetero-structured devices that limits the further performance improvement of solar cells. The flexible engineering of band alignment and interfacial coupling for ultrathin OIHPs can create more possibilities with regard to addressing these challenges. Besides, due to their high optical gain and large absorption coefficient, OIHPs are excellent materials for low-threshold lasing devices [1]. More interestingly, the high-quality factor of the naturally formed microcavity in regular-shaped OIHP nanosheets shows preference to excellent laser sources in flexible optical chips [4,10]. Also, the field of ultrathin OIHP electrical devices, which includes field effect transistors (FETs) and random access memories (RAMs), is fast-moving [7]. Ultrathin OIHPs, according to their flexible structure and multi-functionality, can be easily and highly integrated on various substrates (such as human skin), with great potential in future flexible electronic and optoelectronic fields.

**Figure 2.**
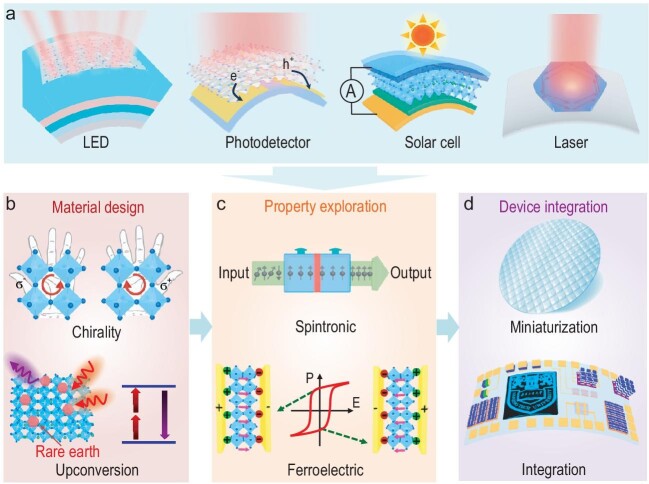
(a) Schematic diagrams of current flexible devices based on OIHPs. Suggested research directions for ultrathin OIHPs in the fields of (b) material design, (c) property exploration and (d) device integration.

Nevertheless, the development of ultrathin OIHPs is still at a very early stage, and more fields are expected to be plumbed in the future. In the field of material design (Fig. 2b), for instance, chiral ultrathin OIHPs can be obtained by introducing the chiral organic ligands into the layered hybrid perovskite frameworks, which is promising in life science, materials science, and spintronic and optoelectronic device applications. Upconversion luminescence properties, which can be achieved by doping rare earth elements, are highly expected in wide applications such as lasers, displays, tissue fluorescence imaging and phototherapy. In addition, wafer-scale and arrayed synthesis of ultrathin OIHPs is, without question, of significant importance as a prerequisite for large-scale device application and integration. With reduced dimensionality, the instability of ultrathin OIHPs to the environment, such as humidity, oxygen, etc., is more significant. Therefore, an increased effort is required to address this issue, for instance, in the directions of surface passivation and device encapsulation. On the other side, their high sensitivity can be put to good use in different types of sensors. For property exploration (Fig. 2c), the working mechanism of most current memory devices is based on ion migration. Therefore, more novel devices based on ferromagnetic and ferroelectric OIHPs are expected to emerge, arising from their giant spin-orbit coupling and broken crystal symmetry [1,5]. Last but not least, based on the merits of ultimate thickness, multi-functionality and easy assembly of 2D materials, ultrathin OIHPs are promising in devices of miniaturization and high integration (Fig. 2d).

In short, ultrathin OIHPs, possessing 2D morphology and hybrid components, have great potential for flexible electronics and optoelectronics. Related research is still in its infancy and future research attention is highly desired. Owing to its ultrathin, flexible and easily integrated nature, as well as its fascinating photonic and optoelectronic properties, we believe that ultrathin OIHP will surely be a significant 2D member widely applied in the near future.

## References

[1] Saparov B , Mitzi DB. Chem Rev 2016; 116: 4558–96. 10.1021/acs.chemrev.5b00715 27040120

[2] Dou L , Wong AB, Yu Y et al. Science 2015; 349: 1518–21. 10.1126/science.aac7660 26404831

[3] Leng K , Abdelwahab I, Verzhbitskiy I et al. Nat Mater 2018; 17: 908–14. 10.1038/s41563-018-0164-8 30202109

[4] Sun Y , Yin Y, Pols M et al. Adv Mater 2020; 32: 2002392. 10.1002/adma.202002392 32686130

[5] Leng K , Fu W, Liu Y et al. Nat Rev Mater 2020; 5: 482–500. 10.1038/s41578-020-0185-1

[6] Pan D , Fu Y, Spitha N et al. Nat Nanotechnol 2021; 16: 159–65. 10.1038/s41565-020-00802-2 33257896

[7] Paulus F , Tyznik C, Jurchescu OD et al. Adv Funct Mater 2021; 31: 2101029. 10.1002/adfm.202101029

[8] Lim KG , Han TH, Lee TW. Energy Environ Sci 2021; 14: 2009–35. 10.1039/D0EE02996C

[9] Jing H , Peng R, Ma RM et al. Nano Lett 2020; 20: 7144–51. 10.1021/acs.nanolett.0c02468 32941049

[10] Zhang Q , Shang Q, Su R et al. Nano Lett 2021; 21: 1903–14. 10.1021/acs.nanolett.0c03593 33435686

